# Intraperitoneal prophylactic drain after pancreaticoduodenectomy: an Italian survey

**DOI:** 10.1007/s13304-024-01836-0

**Published:** 2024-04-25

**Authors:** Claudio Ricci, Nicolò Pecorelli, Alessandro Esposito, Giovanni Capretti, Stefano Partelli, Giovanni Butturini, Ugo Boggi, Alessandro Cucchetti, Alessandro Zerbi, Roberto Salvia, Massimo Falconi, Pan-Drain study group, Laura Alberici, Francesca Aleotti, Sergio Alfieri, Marco Angrisani, Alessandro Anselmo, Elisa Bannone, Matteo Barabino, Giulio Belfiori, Andrea Belli, Giulio Belli, Chiara Bonatti, Gianluca Borgia, Lucio Caccamo, Donata Campra, Damiano Caputo, Riccardo Casadei, Matteo Cescon, Davide Citterio, Ettore Colangelo, Michele Colledan, Roberto Coppola, Stefano Crippa, Tommaso Dall’Olio, Luciano De Carlis, Donato De Giorgi, Raffaele De Luca, Antonella Del Vecchio, Raffaele Della Valle, Fabrizio Di Benedetto, Armando Di Dato, Stefano Di Domenico, Giovanni Di Meo, Pierluigi Di Sebastiano, Maria Ettorre Giuseppe, Alessandro Fogliati, Antonio Frena, Francesco Gavazzi, Batignani Giacomo, Luca Giannotti, Felice Giuliante, Gianluca Grazi, Tommaso Grottola, Salvatore Gruttadauria, Carlo Ingaldi, Frigerio Isabella, Francesco Izzo, Giuliano La Barba, Serena Langella, Gabriella Lionetto, Raffaele Lombardi, Lorenzo Maganuco, Laura Maggino, Giuseppe Malleo, Lorenzo Manzini, Giovanni Marchegiani, Alessio Marchetti, Stefano Marcucci, Marco Massani, Laura Mastrangelo, Vincenzo Mazzaferro, Michele Mazzola, Riccardo Memeo, Caterina Milanetto Anna, Federico Mocchegiani, Luca Moraldi, Francesco Moro, Niccolò Napoli, Gennaro Nappo, Bruno Nardo, Alberto Pacilio Carlo, Salvatore Paiella, Davide Papis, Alberto Patriti, Damiano Patrono, Enrico Prosperi, Silvana Puglisi, Marco Ramera, Matteo Ravaioli, Aldo Rocca, Andrea Ruzzente, Luca Sacco, Grazia Scialantrone, Matteo Serenari, Domenico Tamburrino, Bruna Tatani, Roberto Troisi, Luigi Veneroni, Marco Vivarelli, Matteo Zanello, Giacomo Zanus, Costanza Zingaretti Caterina, Andrea Zironda

**Affiliations:** 1 Department of Internal Medicine and Surgery (DIMEC), Alma Mater Studiorum, University of Bologna, Bologna, Italy; 2 Division of Pancreatic Surgery and Transplantation, Pancreas Translational and Clinical Research Center, San Raffaele Scientific Institute, Milan, Italy; 3 “Vita-Salute” San Raffaele University, Milan, Italy; 4 General and Pancreatic Surgery Department, The Pancreas Institute-University of Verona Hospital Trust, Verona, Italy; 5 Pancreatic Surgery Unit, Humanitas Clinical and Research Center-IRCCS, Via Manzoni 56, Rozzano, Milan Italy; 6 Department of Biomedical Sciences, Humanitas University, Via Rita Levi Montalcini 4, Pieve Emanuele, Milan, Italy; 7 Surgical Department, HPB Unit Pederzoli Hospital, Peschiera Del Garda, Italy; 8 Division of General and Transplant Surgery, University of Pisa, Pisa, Italy; 9 Morgagni e Pierantoni Hospital, Forlì, Italy; 10 Surgical Taskforce of Italian Association for the Study of the Pancreas, Roma, Italy

**Keywords:** Drainage, Pancreatectomy, Survey, Questionnaire, Regret

## Abstract

Intraperitoneal prophylactic drain (IPD) use in pancreaticoduodenectomy (PD) is still controversial. A survey was designed to investigate surgeons’ use of IPD in PD patients through 23 questions and one clinical vignette. For the clinical scenario, respondents were asked to report their regret of omission and commission regarding the use of IPD elicited on a scale between 0 (no regret) and 100 (maximum regret). The threshold model and a multilevel mixed regression were applied. One hundred three (97.2%) respondents confirmed using at least two IPDs. The median regret due to the omission of IPD was 84 (67–100, IQR). The median regret due to the commission of IPD was 10 (3.5–20, IQR). The CR-POPF probability threshold at which drainage omission was the less regrettable choice was 3% (1–50, IQR). The threshold was lower for those surgeons who performed minimally invasive PD (*P* = 0.048), adopted late removal (*P* = 0.002), perceived FRS able to predict the risk (*P* = 0.006), and IPD able to avoid relaparotomy *P* = 0.036). Drain management policies after PD remain heterogeneous among surgeons. The regret model suggested that IPD omission could be performed in low-risk patients.

**Supplementary Information:**

The online version contains supplementary material available at 10.1007/s13304-024-01836-0.

## Introduction

Postoperative pancreatic fistula (POPF) represents the major problem of pancreatic resections, increasing patient morbidity and mortality [1]. For decades, the use of intraperitoneal prophylactic drain (IPD) has been considered by pancreatic surgeons as one of the most important strategies to mitigate the negative effect of clinically relevant POPF (CR-POPF) [2]. The IPD could allow early recognition of POPF and POPF-related complications [3], such as post-pancreatectomy hemorrhage (PPH) [4]. Moreover, IPD could mitigate the negative consequences of CR-POPF by evacuating pancreatic, biliary, enteric juice, and blood from the peritoneal cavity early [3]. However, several issues about IPD use and management remain under debate. Indeed, the dogma of routine IPD use was challenged, especially in low-risk pancreatic remnants, and several randomized studies reported similar complication rates when IPD was omitted [5–8]. Second, the timing of removal was recently investigated, hypothesizing that early removal could be safe [9–13]. Third, the use of active suction was recently investigated, suggesting that the type of drainage system does not influence the development of POPF [14]. Despite the availability of high-quality evidence, the use and management of IPD drain remain heterogeneous, even in high-volume centers [15]. Indeed, it seems that the adoption of modern drain policies, such as IPD omission or early removal, has been very slow by pancreatic surgeons despite the results of RCTs [15]. The present survey was designed to investigate the attitude of pancreatic surgeons in the Italian community toward the use of IPD. Additionally, the regret-based decision model was applied. Regret models were beneficial when medical choices could produce uncertain outcomes. Using the physician's emotional intelligence (“anticipated regret”) elicited by one or more clinical scenarios, it is possible to optimize decision-making by adopting the therapeutic strategy, which implies lesser regret in case of a wrong choice.

## Materials and methods

### Survey

An online survey designed using the online platform Survey Planet® was sent in June 2022 to the Italian community of pancreatic surgeons. Particularly, surgeons affiliated with the Italian Association for the Study of the Pancreas (AISP) and the Italian Association of Hepato-biliary-pancreatic Surgery (AICEP) were contacted. A link to the survey was forwarded using the official email address, the official Twitter and Facebook accounts of AISP, and the official WhatsApp channel of AICEP.

The survey was anonymous, but participants were asked to send an e-mail confirming their participation. All responses were mandatory, and each answer could not be subsequently modified to avoid bias. A selected panel of expert pancreatic surgeons from the Surgical Taskforce of Italian Association for the Study of the Pancreas prepared 23 queries about the use of drainage in PD: 14 multiple choice, 4 visual analog scales, and three open questions (Supplementary file). We collected general information about the participants (gender, age) and their professional level (resident, fellow, or expert surgeon). Also, information about the clinical practice setting was collected: country, institutional volume of pancreatic resection, and, if present, other types of institutional surgical activities (colorectal resection, liver resection, upper gastro-intestinal, or others). Subsequently, we asked the following questions: (i) routine use of Fistula Risk Score (FRS) according to Callery [15]; (ii) number and type of drains used; (iii) timing and indications for drain removal; and iv) motivations behind individual choices using a visual analogue scale (0–10). The study followed the COREQ standards for reporting qualitative research [16]. Ethical approval was not sought for the present study because of its survey nature.

### Regret model

At the end of the survey, a clinical hypothetical vignette was presented to participants to measure their regret when choosing drain placement. The clinical case included a 67-year-old patient with pancreatic head adenocarcinoma, in excellent general conditions, who underwent standard PD with firm pancreatic stump and dilated main pancreatic duct (> 5 mm); intraoperative blood loss was between 400 and 700 mL. The Trudeau catalog [17] (scenario two) was used to relate the FRS (in this case, equal to 1 point) with the CR-POPF risk of 3.6%.

Based on their knowledge, experience, and preference, pancreatic surgeons were asked to elicit their personal regret due to the loss of opportunity of CR-POPF mitigation if the drain was not placed, as well as the regret following the placement of a useless IPD. Thus, the *regret of omission* was here represented by the regret felt by the surgeon who omitted the IPD in a patient who otherwise may have benefited from the drainage in case of CR-POPF occurrence. On the other hand, the *regret of the commission* referred to the regret felt by the surgeon who decided to place an IPD, resulting in useless action because the patients did not develop CR-POPF.

The regret of omission was measured through the following question: *“How would you rate the level of your regret, on a scale of 0 to 100 (0* = *no regret, 100* = *maximum regret) if you decided NOT to place an intraperitoneal prophylactic drain and the patient developed after PD a clinically relevant POPF requiring CT-percutaneous drainage?”*. Regret of the commission was elicited as follows: *“How would you rate the level of your regret, on a scale of 0 to 100 (0* = *no regret, 100* = *maximum regret) if you decided to place an intraperitoneal prophylactic drain after PD, and the patient experienced regular postoperative course without clinically relevant POPF?”*.

In the regret model, Mt represents the POPF threshold at which regret of omission equals the regret of commission: Mt = (1 / [1 + (regret of omission/regret of commission)]) × 100 [18]. In other words, Mt is the probability of clinically relevant POPF at which we are indifferent between two management strategies. If the expected CR-POPF rate is above the threshold, the regret of not placing IPD (omission) will be larger than the regret of placing them (commission). Hence, we should place IPD to minimize regret.

### Statistical analysis

Frequencies and percentages were used to describe categorical data. For continuous measures, mean, standard deviation (SD), median, and interquartile (IQR) ranges were used for continuous values. Age, gender, professional level, hospital type, the main activity of the surgical unit, implementation of minimally-invasive PD (MIPD), type and number of drainage, FRS use, the timing for drain removal, tailored strategy for the low and high-risk pancreatic remnant, perceived importance of FRS, closed system, drain mobilization, drain placement in preventing POPF grade B and C were tested in predicting regret of omission, commission and CR-POPF threshold. For these analyses, multilevel multivariate mixed-effects models were used. In these models, the geographic area of the participants was considered fixed because the study was not interested in regional differences. In other words, the total regression line represents the average Italian centers, independently from geographic origin. The effect of covariates was measured, reporting the coefficient and SE. Post-estimation mean regrets and threshold were calculated for each category. A *P* value < 0.05 indicates a non-negligible effect on the regrets or threshold. Statistical analyses were performed with Stata (Stata Statistical Software: Release 15, StataCorp, LLC, College Station, TX).

## Results

### Participants

The survey was released on July 08, 2022, and was closed on August 31, 2022. One hundred six surgeons completed the online questionnaire. At the time of the survey, 143 surgeons were registered in AISP and AICEP. The engagement rate was 74.1%. In Table 1, the general information of respondents is shown. The median age of respondents was 46 years (36–57). 88.7% of respondents were attending surgeons, while 11.3% were residents or fellows. Most surgeons (71.7%) worked in hospitals located in Northern Italy (Lombardy, Emilia-Romagna, Veneto, Piedmont Trentino South-Tyrol, Friuli-Venezia Giulia, and Liguria). The remaining 33% were located in Central (Lazio, Tuscany, and Marche) or Southern Italy (Puglia, Campania, Abruzzi, Basilicata, Calabria, and Sicily) of Italy. Most worked in public academic (51.9%) or non-academic (23.6%) hospitals. The remaining 26 participants worked in private academic (17.9%) or private non-academic (6.6%) hospitals. Most participants (66%) worked in high-volume (> 30 pancreatic resections yearly) centers, while 24.5% and 17.9% were in medium and low-volume hospitals, respectively. Regarding the main surgical activity of their division, 35.9% answered hepato-biliary, 30.2% answered pancreatic, and 5.7% answered colorectal resections. Only 28.3% declared to work in a division where all sub-specialties mentioned above were equally represented. Only 33.9% of surgeons declared to perform MIPD.

**Table 1:** Characteristics of 106 participants

Characteristics of participants	*N* (%) or median (IQR)
Sex	
Female	18 (17)
Male	88 (83)
Age, years	46 (36–57)
Professional Level	
Resident/Fellow	12 (11.3)
Attending	94 (88.7)
Geographic area	
North of Italy	71 (67)
Center of Italy	16 (15.1)
South of Italy	19 (17.9)
Hospital type	
Public, non-academic	25 (23.6)
Private, non-academic	7 (6.6)
Private, academic	19 (17.9)
Public, academic	55 (51.9)
Hospital volume of pancreatic resection, yearly	
< 10	5 (4.7)
11–20	14 (13.2)
21–30	17 (16)
31–40	9 (8.5)
41–50	10 (9.4)
51–100	25 (23.6)
> 100	26 (24.5)
Type of surgical unit	
Colo-rectal	6 (5.7)
Hepato-biliary	38 (35.9)
Pancreatic	32 (30.2)
General surgery, including all sub-specialties	30 (28.3)
MIPD	
No	70 (66.1%)
Yes	36 (33.9%)

*IQR* interquartile range, *MIPD* Minimally invasive pancreaticoduodenectomy

### Use and management of drainage

The use and management of drains are reported in Table 2. Most surgeons (49.1%) declared using an Easy Flow or Penrose-type passive drain. The second most used drainage (33%) was a closed system (Jackson-Pratt or Blake drainage) with or without active suction. Robison drainage (i.e., silicone round drain with closed system) was used only by 15.1% of participants. Almost all respondents place two or more drains (97.2%) after PD. Only 24.5% of surgeons remove the IPD within the third POD despite the criteria for early removal being satisfied. Two-thirds of the respondents (66%) routinely use FRS. The median perceived importance of FRS in predicting CR-POPF was 2 (0–6, IQR); the median perceived importance of a closed system in predicting CR-POPF grade B was 3 (0–5, IQR); the perceived importance of drain mobilization in mitigating CR-POPF grade B was 5 (2–7, IQR); the perceived importance of drain in preventing CR-POPF grade C was 6 (3–8, IQR).

**Table 2:** Survey results about the use of drains after pancreaticoduodenectomy

Characteristics	*n* (%) or median (IQR)
Type of drainage	
Easy flow/Penrose drain	52 (49.1)
Robinson silicone round drain	16 (15.1)
Jackson-Pratt or Blake drainage, with closed system and active suction	19 (17.9)
Jackson-Pratt or Blake drainage, with closed system and without active suction	18 (17)
Others	1 (0.9)
Number of drainages routinely used	
One	3 (2.8)
Two	72 (67.9)
More than two	31 (29.3)
Change of strategy in high-risk pancreatic remnant	
No	86 (81.1)
Yes	20 (18.9)
Change of strategy in low-risk pancreatic remnant	
No	91 (85.9)
Yes	15 (14.2)
Timing of removal^a^	
≤ III POD	26 (24.5)
IV–V POD	44 (41.5)
VI–VII POD	30 (28.3)
> VII POD	6 (5.7)
FRS use	
No	36 (34)
Yes	70 (66)
Perceived importance of FRS in predicting CR-POPF^b^	2 (0–6)
Perceived importance of closed system in preventing CR-POPF grade B^b^	3 (0–5)
Perceived importance of drain mobilization in mitigating CR-POPF grade B^b^	5 (2–7)
Perceived importance of drain in preventing a re-intervention^b^	6 (3–8)

*IQR* interquartile range, *FRS* Fistula Risk Score, *POD* Postoperative days, *FRS* Fistula Risk Score, *CR-POPF* Clinically Relevant Postoperative Pancreatic Fistula

^a^In case of low drain fluid amylase concentration, patient in good clinical conditions and absence of suspicious fluid in the drain

^b^Scale from 0 to 10

A change of strategy in low-risk pancreatic stumps was declared by 14.2% of respondents, reducing the number of drains (5.6%) or not placing any (4.7%). A change of strategy in high-risk pancreatic remnants was declared by 18.9% of respondents, increasing the number of drains (10.4%), changing the type (4.7%), or both (3.8%).

### Regret analysis

**Fig. 1:**
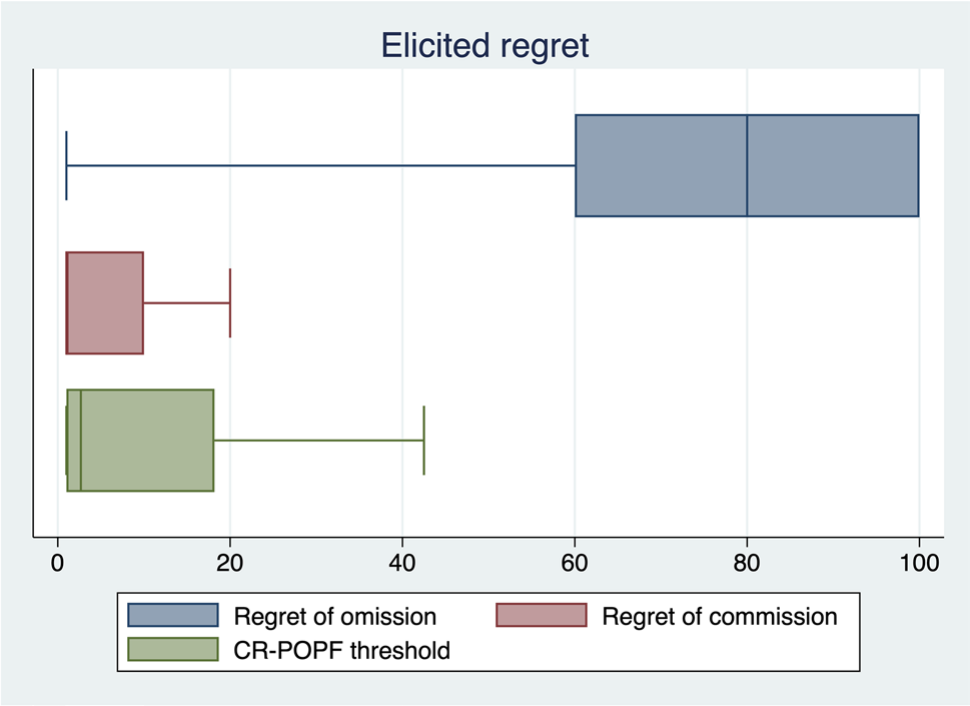
Box plots reporting regret of omission, commission, and CR-POPF threshold in the clinical vignette presented to the 106 respondents

**Fig. 2:**
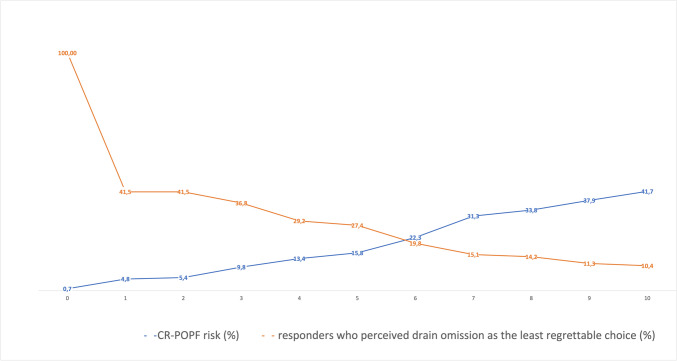
Percentage of responders who consider the IPD omission as the least regrettable choice based on the risk of CR-POPF. The x-axis represents the Fistula Risk Score categories (FRS); the blue line reports the risk of CR-POPF related to each category of FRS according to Trudeau et al. [22]; the orange line reports the percentage of responders who perceived the IPD omission as the least regrettable choice for the related risk of CR-POPF

Regret of omission, commission, and thresholds are reported in Fig. 1. The mean regret of omission was 73 (± 31, SD), with a median of 80 (60–100, IQR). The mean regret of the commission was 10 (± 16.8, SD) with a median of 1 (1–10). The mean CR-POPF risk probability threshold at which drainage omission was the less regrettable choice was consequently 12(± 18) % with a median of 3% (1–18%, IQR). In Fig. 2, we reported the percentage of responders who perceived IPD omission as the least regrettable choice for each value of FRS and related probability of CR-POPF.

Multilevel effect multivariate regressions are reported in Supplementary Table 1, while the estimated mean of regrets and threshold was reported in Table 3**.** Age, gender, professional level, hospital type, active suction preference, custom to change strategy based on the risk, and perceived importance of drain mobilization did not affect regrets and threshold for CR-POPF. Responders working in high-volume hospitals had significantly lower mean regret of omission than those working in low-volume hospitals (77 ± 19 vs. 71 ± 18; *P* < 0.001).

**Table 3:** Postestimation values after multilevel mixed-effects multivariate regression

Covariates^a,b^	Regret of omission		Regret of commission		Risk threshold for CR-POPF^c^	
	Mean ± SD	*P* value	Mean ± SD	*P* value	Mean ± SD	*P* value
Hospital volume						
Low–Medium	77 ± 19	< 0.001	12 ± 9	< 0.001	12 ± 7	0.299
High	71 ± 18		7 ± 7		13 ± 7	
Prominent activity of surgical unit						
Colorectal	68 ± 21	Ref	24 ± 8	Ref	23 ± 5	Ref
Hepato-biliary	81 ± 15	0.405	11 ± 6	0.542	12 ± 6	0.781
Pancreatic	72 ± 19	0.021	10 ± 6	0.727	13 ± 7	0.722
General surgery, including all sub-specialties	67 ± 17	0.483	4 ± 7	0.075	11 ± 7	0.467
MIPD						
No	75 ± 20	0.224	9 ± 8	0.741	13 ± 7	0.048
Yes	69 ± 16		10 ± 7		12 ± 7	
Type of drain						
Robinson, Jackson-Pratt, or Blake	76 ± 17	0.676	12 ± 8	< 0.001	14 ± 6	0.578
Easy Flow/Penrose	70 ± 20		7 ± 6		11 ± 8	
Type of system						
Open	75 ± 20	0.546	6 ± 6	< 0.001	11 ± 7	0.004
Close	69 ± 16		14 ± 7		15 ± 6	
Number of drains						
One or two	67 ± 16	0.004	10 ± 7	0.516	14 ± 7	0.199
More than two	90 ± 13		8 ± 9		8 ± 6	
FRS use						
No	88 ± 11	0.001	8 ± 3	0.659	9 ± 6	0.211
Yes	66 ± 17		10 ± 8		15 ± 7	
Timing for drain removal						
Early	60 ± 18	0.906	7 ± 9	0.355	15 ± 9	0.002
Late	78 ± 17		10 ± 7		12 ± 6	
Importance of FRS in predicting CR-POPF (VAS scale)^d^						
≤ 2	80 ± 16	< 0.001	7 ± 9	0.011	10 ± 6	0.006
> 2	63 ± 18		13 ± 8		17 ± 7	
Importance of closed system in preventing grade B CR-POPF (VAS scale)^d^						
≤ 3	71 ± 19	0.054	6 ± 6	< 0.001	12 ± 7	0.201
> 3	76 ± 17		14 ± 7		13 ± 7	
Importance of drain in preventing re-intervention (VAS scale)^d^						
≤ 6	68 ± 18	< 0.001	11 ± 7	< 0.001	14 ± 7	0.036
> 6	79 ± 18		7 ± 8		10 ± 7	

*FRS* Fistula Risk Score, *CR-POPF* Clinically Relevant Postoperative Pancreatic Fistula

^a^We report only covariates significantly affecting regret of omission, commission, or threshold

^b^Age, gender, professional level, hospital type, active suction preference, custom to change strategy based on the risk, and perceived importance of drain mobilization did not affect regrets and threshold for CR-POPF

^c^The threshold indicates the CR-POPF risk rate at which the drain omission is the least regrettable choice, calculated with FRS

^d^The median values of visual analogic scale (VAS) are used to dichotomize the variables

Compared with colorectal surgeons, participants who work in a specialized pancreas unit had an increased mean regret of omission (72 ± 19 vs. 68 ± 21; *P* = 0.021). However, mean regrets of commission and the final threshold for CR-POPF remain unaffected by the prominent activity of the surgical unit. Comparing MIPD and non-MIPD surgeons, the mean final threshold for CR-POPF was 13 ± 7% vs. 12 ± 7% (*P* = 0.048), respectively. Surgeons who preferred Easy Flow or Penrose drains (7 ± 6) had a significantly lower (*P* < 0.001) mean regret of commission than those who preferred Robinson, Jackson-Pratt, or Blake (12 ± 8) drains. Participants who used a closed system had higher threshold for CR-POPF than those adopted open systems (15 ± 6% vs. 11 ± 7; *P* = 0.004). Obviously, the answerer who used more than two drains after PD experienced a higher regret of omission than those who placed only one or two drains (90 ± 13 vs. 67 ± 16; *P* = 0.004). The survey participants who used the FRS score had a lower mean regret of omission than those who did not use this prediction system (88 ± 11 vs. 66 ± 17; *P* = 0.001). The responders who routinely removed the drain within POD 3 had a superior mean threshold (12 ± 7 vs. 15 ± 9; *P* = 0.002). The higher the perceived importance of FRS in predicting CR-POPF, the lower the regret of omission (*P* ≤ 0.001) and CR-POPF threshold (1.1 ± 0.4; *P* = 0.006). On the contrary, increasing the perceived importance of FRS, the regret of the commission was slightly higher (*P* = 0.011). The perceived importance of the closed system in preventing Grade B CR-POPF slightly influenced the regret of the commission (*P* < 0.001) but not the regret of omission and CR-POPF threshold. The perceived importance of drain placement in preventing Grade C CR-POPF influenced both regrets and threshold: the higher the perceived importance, the higher the regret of omission (*P* < 0.001); the higher the perceived importance, the lower the regrets of commission (*P* < 0.001) and CR-POPF threshold (*P* = 0.036).

## Discussion

The present survey demonstrated that Italian pancreatic surgeons routinely use IPD after PD, as 98% of participants declared placing two or more drains at the end of surgery. Only a minority of interviewed surgeons reported a change in perioperative drain strategy based on the intraoperative characteristics of the pancreatic remnant and the relative pancreatic fistula risk score, suggesting a preference for a standard and consistent drain policy rather than a selective POPF mitigation strategy. In addition, our work highlighted the large heterogeneity in drain type preference and postoperative drain management among the Italian community of pancreatic surgeons.

This survey outlines a “real-life” scenario in which the IPD is deemed as a necessary tool for safely monitoring and managing the postoperative course after PD. This attitude is confirmed by the reluctance to remove the IPD early, even when patients were clinically well, drain fluid amylase concentration was low, and the quality of the fluid was not suspicious. Indeed, only one out of four survey participants adopts an early drain removal policy in their practice. Surprisingly, more than 30% of surgeons remove the IPD very late, after POD5, even when the postoperative course was uneventful. These data are in contrast with the available evidence. In fact, a recent meta-analysis of RCTs showed that IPD omission after pancreatic resection is a safe alternative to their routine use in low-risk scenarios [2]. Moreover, at least five recent RCTs [9–13] supported adopting early removal in patients with low risk for CR-POPF and regular postoperative stay. Indeed, in recent recommendations from the Enhanced Recovery After Surgery (ERAS) Society for patients undergoing PD, the level of evidence in favor of selective IPD omission and early removal was moderate and high, respectively [19]. It may appear surprising that one of the cornerstones of the ERAS philosophy finds a high resistance in the pancreatic surgeon community. Still, it is no secret that surgical traditions are the most challenging to abandon.

The present survey suggested that the dogma of mandatory drainage persists, confirming the results of a previous survey by Pergolini et al. [20], including 42 expert pancreatic surgeons. However, in our survey, we also investigated the reasons for resistance to selective IPD omission using the “regret theory” approach. The regret methodology gives a scientific value to emotional intelligence: a physician making a nonrepeatable decision under uncertainty (e.g., omission or not of therapy) could experience regret in case of a negative result. This regret can be measured and used to optimize the choices [21]. Measuring the regret of omission and commission in a prespecified scenario allows us to calculate the acceptable probability threshold for a negative event, at which the omission of therapy is the least regrettable choice.

In the current survey, we asked participants to anticipate the regret, using a clinical case of a patient undergoing PD with an expected low risk of CR-POPF. By asking the responders to elicit both regrets (commission and omission) in a real-life scenario, we captured all different shades of the emotional intelligence of interviewed surgeons without imposing pre-concepts about the drain-less policy. Moreover, a low-risk scenario was chosen in intermediate and high-risk scenarios of CR-POPF; the omission of drainage is generally not accepted by pancreatic surgeons. For this reason, the drain-less approach could not reflect a real-life problem when the risk of CR-POPF is not low.

As expected, the median regret of omission was very high, while the regret of the commission was very low, confirming the pancreatic surgeons' unwillingness to accept the selective IPD omission. The median value of CR-POPF risk probability, at which the least regrettable choice was the IPD omission, was 3%. This result may appear to strengthen IPD omissions as a definitive choice in low-risk scenarios. Using the FRS catalog of Trudeau et al. [16] that reported granular data about FRS scenarios occurrence and CR-POPF risk, it is possible to estimate how many patients could be managed without drainage based on the emotional intelligence of pancreatic surgeons. In fact, it is surprising that nearly 15% of patients who underwent PD could have a risk of CR-POPF equal to or inferior to 3%. For this reason, based on regret theory, nearly one patient out of six/seven could be managed with selective IPD omission despite worldwide reluctance. We observed some interesting findings by analyzing factors related to the CR-POPF threshold and regrets. Regret of omission was reduced in centers at high volume for pancreatic surgery (> 30 pancreatic resections/year). Surgeons who work in high-volume hospitals can rely on key expertise and resources, such as interventional radiology [22] and operative endoscopy [23], that can manage peri-anastomotic fluid collections and other life-threatening complications related to POPF (i.e., post-pancreatectomy hemorrhage). At the same time, dedicated pancreatic surgeons perceived and feared, more than other surgeons, the devastating potential effects of undrained CR-POPF. Interestingly, the regret of omission was reduced in those surgeons who looked at FRS as trustworthy in predicting CR-POPF. Thus, in the presence of a well-established and valid tool to anticipate CR-POPF risk, the attitude of surgeons in adopting selective IPD omission is enhanced. In other words, the reluctance to adopt IPD omission seems more related to the lack of confidence in the risk score system to predict the CR-POPF risk than to an absolute refusal of this strategy. Indeed, when surgeons were more confident using FRS, the IPD omission represented the least regrettable choice for CR-POPF probability higher than 3%. However, the problem seems to be the overall low confidence in FRS’s ability. In fact, the median perceived importance is very low (2 out of 10 on the VAS scale) despite the amount of literature available in favor of this score [15, 16]. Also, the role of IPD in preventing reintervention is generally overestimated, and the higher the perceived importance of drainage in preventing grade C CR-POPF, the higher the regret of omission. In fact, the CR-POPF threshold, at which IPD omission is the least regrettable choice, was lower than 3% for the surgeons who overestimated the role of IPD in preventing reintervention. An interesting finding is that minimally invasive PD surgeons have a shallow CR-POPF threshold. In other words, pancreatic surgeons perceive the minimally invasive approach as a procedure more at risk for CR-POPF than open. For this reason, selective IPD omission is often considered a regrettable strategy in very low-risk scenarios. In contrast, surgeons who used a closed drain system seem to adopt IPD omission also in settings with a higher risk of CR-POPF (more than 3%). This may be explained by the theory of retrograde infection supported by those pancreatic surgeons using the closed system; in the event of a small biochemical leak, the presence and persistence of a peripancreatic drain could increase the fistula output and facilitate a retrograde infection, converting it into a CR-POPF [24, 25]. However, a recent non-inferiority trial has demonstrated that more than 60% of bacteria contaminating the drainage fluid after PD were attributable to human gut flora rather than external bacteria [2].

The current study has some limitations. First, the group of survey participants was very heterogeneous, including surgeons performing pancreatic resections in low and high-volume settings in academic and non-academic institutions. Nonetheless, the survey is a snapshot of “real-life” clinical practice in Italian hospitals. Second, each respondent declared their habits based on personal and center experience, which did not necessarily reflect adequate and updated knowledge of the literature available. The third limitation is represented by the fact that we assumed that a single decision-maker is involved in the IPD omission or commission.

In conclusion, this survey demonstrated that, despite the availability of several RCTs and metanalysis about IPD use after PD, a certain reluctance from pancreatic surgeons to abandon the dogmas exists. This reluctance appears related to multiple factors: (i) the shortcomings of available risk prediction tools for CR-POPF; (ii) the uncertainty of outcomes from a minimally invasive approach in PD; (iii) the concern about an increase in the re-laparotomy rate. Generally, an interesting observation was that, despite the amount of literature available encouraging early removal [5] or drainage omission [8] in low-risk patients, the customs of Italian pancreatic surgeons remain very conservative. Nonetheless, evaluating emotional intelligence, which depends on experience and knowledge, can help surgeons understand that selective IPD omission could be the least regrettable choice when the risk of CR-POPF is low. Considering this, it seems very important an educational process to ameliorate the adoption of a tailored drain policy risk based in patients who underwent PD. On the other hand, it is important to underline that patients with intermediate or high risk of CR-POPF largely benefit from drain placement [28]. Further studies are required to achieve progress in solving this issue.

## Supplementary Information

Below is the link to the electronic supplementary material.

Supplementary file1 (DOCX 281 KB)

Supplementary file2 (DOCX 24 KB)

## Data Availability

The data of the survey were available, on request.
