# Correction: Intraperitoneal prophylactic drain after pancreaticoduodenectomy: an Italian survey

**DOI:** 10.1007/s13304-024-01967-4

**Published:** 2024-09-03

**Authors:** Claudio Ricci, Nicolò Pecorelli, Alessandro Esposito, Giovanni Capretti, Stefano Partelli, Giovanni Butturini, Ugo Boggi, Alessandro Cucchetti, Alessandro Zerbi, Roberto Salvia, Massimo Falconi, Pan-Drain study group, Laura Alberici, Francesca Aleotti, Sergio Alfieri, Marco Angrisani, Alessandro Anselmo, Elisa Bannone, Matteo Barabino, Giulio Belfiori, Andrea Belli, Giulio Belli, Chiara Bonatti, Gianluca Borgia, Lucio Caccamo, Donata Campra, Damiano Caputo, Riccardo Casadei, Matteo Cescon, Davide Citterio, Ettore Colangelo, Michele Colledan, Roberto Coppola, Stefano Crippa, Tommaso Dall’Olio, Luciano De Carlis, Donato De Giorgi, Raffaele De Luca, Antonella Del Vecchio, Raffaele Della Valle, Fabrizio Di Benedetto, Armando Di Dato, Stefano Di Domenico, Giovanni Di Meo, Pierluigi Di Sebastiano, Maria Ettorre Giuseppe, Alessandro Fogliati, Antonio Frena, Francesco Gavazzi, Batignani Giacomo, Luca Gianotti, Felice Giuliante, Gianluca Grazi, Tommaso Grottola, Salvatore Gruttadauria, Carlo Ingaldi, Frigerio Isabella, Francesco Izzo, Giuliano La Barba, Serena Langella, Gabriella Lionetto, Raffaele Lombardi, Lorenzo Maganuco, Laura Maggino, Giuseppe Malleo, Lorenzo Manzini, Giovanni Marchegiani, Alessio Marchetti, Stefano Marcucci, Marco Massani, Laura Mastrangelo, Vincenzo Mazzaferro, Michele Mazzola, Riccardo Memeo, Caterina Milanetto Anna, Federico Mocchegiani, Luca Moraldi, Francesco Moro, Niccolò Napoli, Gennaro Nappo, Bruno Nardo, Alberto Pacilio Carlo, Salvatore Paiella, Davide Papis, Alberto Patriti, Damiano Patrono, Enrico Prosperi, Silvana Puglisi, Marco Ramera, Matteo Ravaioli, Aldo Rocca, Andrea Ruzzente, Luca Sacco, Grazia Scialantrone, Matteo Serenari, Domenico Tamburrino, Bruna Tatani, Roberto Troisi, Luigi Veneroni, Marco Vivarelli, Matteo Zanello, Giacomo Zanus, Costanza Zingaretti Caterina, Andrea Zironda

**Affiliations:** 1 Department of Internal Medicine and Surgery (DIMEC), Alma Mater Studiorum, University of Bologna, Bologna, Italy; 2 Division of Pancreatic Surgery and Transplantation, Pancreas Translational and Clinical Research Center, San Raffaele Scientific Institute, Milan, Italy; 3 “Vita-Salute” San Raffaele University, Milan, Italy; 4 General and Pancreatic Surgery Department, The Pancreas Institute-University of Verona Hospital Trust, Verona, Italy; 5 Pancreatic Surgery Unit, Humanitas Clinical and Research Center-IRCCS, Via Manzoni 56, Rozzano, Milan Italy; 6 Department of Biomedical Sciences, Humanitas University, Via Rita Levi Montalcini 4, Pieve Emanuele, Milan, Italy; 7 Surgical Department, HPB Unit Pederzoli Hospital, Peschiera Del Garda, Italy; 8 Division of General and Transplant Surgery, University of Pisa, Pisa, Italy; 9 Morgagni e Pierantoni Hospital, Forlì, Italy; 10 Surgical Taskforce of Italian Association for the Study of the Pancreas, Roma, Italy

**Correction: Updates in Surgery (2024) 76:923–932** 10.1007/s13304-024-01836-0

In this article the author name Luca Gianotti was incorrectly written as Luca Giannotti under the Acknowledgement section.

The original article has been corrected.

