# Supplementary material for: Intraperitoneal prophylactic drain after pancreaticoduodenectomy: an Italian survey
Source: Updates Surg. 2024 Apr 25;76(3):923–32. doi: 10.1007/s13304-024-01836-0 (PMC11130052; doi:10.1007/s13304-024-01836-0)
Supplement: Supplementary file 1 — Supplementary file1 (DOCX 281 KB) [file 13304_2024_1836_MOESM1_ESM.docx]

**Survey**

Q1 E-mail: free field

Q2 Gender

1. Male
2. Female
3. Other

Q3 Professional level

1. Resident
2. Attending
3. Fellow/PhD student

Q4 Hospital type

1. Public, non-academic
2. Private within NHS, non-academic
3. Private, academic
4. Public, academic
5. Private outside NHS, non-academic

Q5 Geographic area: free field

Q6 - In which hospital do you mainly carry out your clinical activity? (specify the official name of hospital and ward): free field

Q7 - How many pancreatic resections were performed in 2021 at your center?

1. <10
2. 11-20
3. 21-30
4. 31-40
5. 41-50
6. 51-100
7. >100

Q8 - How much does pancreatic surgery account for in your department?

1. Less than 10%
2. 11% - 25%
3. 26% - 50%
4. 51% - 75%
5. More than 75%

Q9 - Which of the following definitions would you consider most appropriate if you were to define the main address of the department in which you work?

1. Colorectal Surgery
2. Upper GI Surgery
3. Hepatobiliary Surgery
4. Pancreatic Surgery
5. Other

Q10 - Do you use the Fistula Risk Score (FRS) intraoperative to stratify the risk of pancreatic fistula after DCP?

1. Yes
2. No, but a similar score
3. No

Q11 - In the PD how many drains do you place routinely?

1. 0
2. 1
3. 2
4. 3
5. More than 3

Q12 - In the PD how many drains do you routinely electively devote to pancreatic anastomosis?

a) 0

b) 1

c) more than 1

Q13 - In the PD routinely, what type of drainage do you use for pancreatic anastomosis?

1. Easy flow/Penrose drainage
2. Robinson drainage
3. Jackson-Pratt or Blake drainage, with closed system and active suction
4. Jackson-Pratt or Blake drainage, with closed system and without active suction
5. Others

Q14 - During the PD, in the case of a patient at high risk of pancreatic fistula (soft stump, Wirsung not dilated), does it change its policy on drainage?

1. No
2. Yes, increasing the number of drainages
3. Yes, the type of drainage
4. Yes, the number and type

Q15 - In the PD, in the case of a patient at low risk of pancreatic fistula (hard stump, Wirsung dilated) does it change its policy on drainage?

1. No
2. Yes, omitting the drainage
3. Yes, changing the type of drainage
4. Yes, reducing the number

Q16 - In the case of minimally invasive PD, does it change its drainage policy?

1. Not performed
2. No
3. Yes, increasing the number of drainages
4. Yes, the type of drainage
5. Yes, the number and type

Q17 - Following PD in case of good clinical condition, absence of fever, negative amylase, and non-suspicious appearance of the material, on which postoperative day (GPO) do you proceed to the removal of pancreatic drainage?

1. <= III POD
2. IV-V POD
3. VI-VII POD
4. >VII POD

Q18 - Following PD on a scale from 0 to 10, what value do you think the closed system has in preventing the appearance of a grade B pancreatic fistula?

Q19 - Following PD on a scale from 0 to 10, what value do you think the mobilization of passive drainage has in reducing the duration of grade B pancreatic fistula?

Q20 - Following PD on a scale from 0 to 10, what is the value of drainage in preventing reintervention for pancreatic fistula (grade C)?

Q21 - How much does the FRS (or other similar scores) affect, on a scale from 0 to 10, your decision to place the drains after PD?

**Vignettes**

**Clinical case:**

*67-year-old patient with pancreatic head adenocarcinoma, in excellent general condition, underwent standard PD, firm pancreatic remnant and Wirsung >5 mm. The blood loss is between 400 and 700 mL.*

**Regret of omission**

*“How would you rate the level of your regret, on a scale of 0 to 100 (0 = no regret, 100 = maximum regret) if you decided NOT to place the intraperitoneal prophylactic drain and the patient developed after PD a clinically relevant POPF requiring CT- percutaneous drainage?”*

**

**Regret of commission**

*“How would you rate the level of your regret, on a scale of 0 to 100 (0 = no regret, 100 = maximum regret) if you decided to place the intraperitoneal prophylactic drain and the patient had, after PD, normal postoperative course without clinically relevant POPF?”*

**
