# Supplementary material for: Intraperitoneal prophylactic drain after pancreaticoduodenectomy: an Italian survey
Source: Updates Surg. 2024 Apr 25;76(3):923–32. doi: 10.1007/s13304-024-01836-0 (PMC11130052; doi:10.1007/s13304-024-01836-0)
Supplement: Supplementary file 2 — Supplementary file2 (DOCX 24 KB) [file 13304_2024_1836_MOESM2_ESM.docx]

**Supplementary Table 1 – Multilevel mixed-effects regression evaluating the effect of the covariates on the Regret of omission, commission, and CR-POPF threshold.**

| **Covariates *** | **Regret of omission** | | **Regret of commission** | | **Threshold for CR-POPF ^** | |
| --- | --- | --- | --- | --- | --- | --- |
|  | **Coefficient ± SE** | **p-value** | **Coefficient ± SE** | **p-value** | **Coefficient ± SE** | **P-value** |
| **Age** (for each year) | 0.1 ± 0.3 | 0.646 | -0.1 ± -0.1 | 0.503 | -0.5 ± 5.4 | 0.928 |
| **Gender** (Male vs. Female) | -5.7 ± 5.1 | 0.264 | 4.8 ± 4.4 | 0.279 | -0.1 ± -0.1 | 0.738 |
| **Professional Level** (Resident vs. Trainee) | -0.6 ± 8.9 | 0.945 | -0.5 ± 2.1 | 0.806 | 2.7 ± 2.5 | 0.301 |
| **Hospital** **type** (Teaching vs. non-teaching) | -8.0 ± 4.9 | 0.107 | -0.3 ± 2.8 | 0.917 | 1.5 ± 2.9 | 0.614 |
| **Hospital type** (Public vs. Private) | -2.1 ± 3.4 | 0.527 | -1.5 ± 4.7 | 0.745 | -4.3 ± 3.9 | 0.270 |
| **Hospital volume** (Low-Medium vs. High) | -4.5 ± 0.9 | <0.001 | -1.7 ± 0.1 | <0.001 | 1.5 ± 1.5 | 0.299 |
| **Prominent activity of surgical unit**  Colorectal  Hepato-biliary  Pancreatic  General surgery, including all sub-specialties | 1.0(referent)  8.6 ± 8.5  16.9 ± 7.3  5.1 ± 7.2 | 0.405  0.021  0.483 | 1.0(referent)  -7.3 ± 11.9  -4.8 ± 13.7  -14.9 ± 8.4 | 0.542  0.727  0.075 | 1.0(referent)  -4.3 ± 15.2  -4.9 ± 13.8  -8.8 ± 12.1 | 0.781  0.722  0.467 |
| **MIPD** (No vs. Yes) | 5.5 ± 4.5 | 0.224 | 1.3 ± 3.9 | 0.741 | - 3.0 ± 1.6 | 0.048 |
| **Type of drain** (Robinson, Jackson-Pratt, or Blake vs. Easy Flow/Penrose) | -6.8 ± 16.3 | 0.676 | -0.5 ± 0.1 | <0.001 | -2.8 ± 5.1 | 0.578 |
| **Type of system** (Open vs. Close) | -4.5 ± 7.6 | 0.546 | 5.1 ± 1.1 | <0.001 | 6.2 ± 2.2 | 0.004 |
| **Active suction** (No vs. Yes) | 3.5 ± 6.1 | 0.558 | -0.5 ± 5.5 | 0.923 | -1.6 ± 1.3 | 0.216 |
| **Number of drain** (One or two vs. more than two) | 13.6 ± 4.7 | 0.004 | -0.8 ± 1.3 | 0.516 | -1.9 ± 1.5 | 0.199 |
| **FRS use** (No vs. Yes) | -12.1 ± 3.7 | 0.001 | 2.7 ± 6.1 | 0.659 | 7.2 ± 5.8 | 0.211 |
| **Change strategy in low-risk pancreatic remnant** (No vs. Yes) | -5.1 ± 8.3 | 0.536 | 0.9 ± 2.5 | 0.725 | 0.2 ± 1.5 | 0.905 |
| **Change strategy in high-risk pancreatic remnant** (No vs. Yes) | -1.8 ± 3.9 | 0.657 | -2.3 ±1.2 | 0.056 | 2.1 ±2.6 | 0.444 |
| **Timing for drain removal** (early vs. late) | 0.5 ± 4.6 | 0.906 | 4.1 ± 4.4 | 0.355 | -0.5 ± 3.1 | 0.002 |
| **Importance of FRS in predicting CR-POPF** (for each point) | -2.6 ± 1.1 | <0.001 | 0.4 ± 0.2 | 0.011 | 1.1 ± 0.4 | 0.006 |
| **Importance of closed system in preventing grade B CR-POPF** (for each point) | 1.5 ± 0.7 | 0.054 | 0.6 ± 0.2 | <0.001 | -0.7 ± 0.5 | 0.201 |
| **Importance of drain mobilization in mitigating grade B CR-POPF** (for each point) | 0.3 ± 0.6 | 0.664 | 0.5 ± 0.4 | 0.182 | 0.3 ± 0.4 | 0.337 |
| **Importance of drain in preventing re-intervention** (for each point) | 2.1 ± 0.5 | <0.001 | -1.1 ± 0.2 | <0.001 | -0.5 ± 0.3 | 0.036 |

**Legend**: *= geographic area was included in all models as fixed effect; ^= The CR-POPF risk rate at which the drain omission is the least regrettable choice, calculated with FRS; FRS= Fistula Risk Score; CR-POPF= Clinically Relevant Postoperative Pancreatic Fistula.
